# Children’s Mental Health During the First Two Years of the COVID-19 Pandemic: Burden, Risk Factors and Posttraumatic Growth – A Mixed-Methods Parents’ Perspective

**DOI:** 10.3389/fpsyg.2022.901205

**Published:** 2022-06-02

**Authors:** Anna Wenter, Maximilian Schickl, Kathrin Sevecke, Barbara Juen, Silvia Exenberger

**Affiliations:** ^1^Department of Psychology, University of Innsbruck, Innsbruck, Austria; ^2^Department of Child and Adolescent Psychiatry, Medical University of Innsbruck, Innsbruck, Austria; ^3^Department of Child and Adolescent Psychiatry, Psychotherapy and Psychosomatics, Tirol Kliniken, Innsbruck, Austria

**Keywords:** COVID-19, mental health, psychiatric symptoms, quality of life, risk factors, threat experience, posttraumatic growth, children

## Abstract

The COVID-19 pandemic and the accompanying containment measures such as physical distancing and school closures led to major changes in children’s everyday lives. By means of a mixed-methods study, the “Tyrolean COVID-19 Children’s Study” investigated the effects of the pandemic and factors influencing mental health and health-related quality of life of North Tyrolean (Austria) and South Tyrolean (Italy) children aged 3–13 years. Parents filled out *N* = 2,691 online questionnaires (951 preschool children: 3–6 years; 1,740 schoolchildren: 7–13 years) at four measurement time points (March 2020, December 2020, June 2021, December 2021). For both age groups, children’s mental health outcomes (internalising problems, posttraumatic stress symptoms) were worse in December 2021 (t4) than children’s mental health outcomes in March 2020 (t1). With regard to aggressive behaviour, this difference was only found among schoolchildren. Thematic analysis of an open ended, written question revealed the following positive changes in children during the Corona crisis: (1) the importance of intra- and extra-familial relationships, (2) new competences and experiences, (3) values and virtues, (4) use of time, and (5) family strength. Using multilevel modelling, threat experience, economic disruption, and perceived posttraumatic growth were shown to be the strongest predictors of all outcomes. Additionally, male gender was shown to be a predictor of aggressive behaviour. In terms of age, schoolchildren showed more internalising problems, aggressive behaviour, and threat experience than preschool children. With regard to time, parents in December 2021 reported more threat experience in older children and less perceived posttraumatic growth in both older and younger children, than parents at the beginning of the pandemic. Targeted support for vulnerable children may prevent longer-term development of psychopathologies and contribute to society’s psychosocial resilience in the current COVID-19 pandemic. Moreover, sustainable promotion of children’s posttraumatic growth can also contribute to children’s mental health and could even offer a chance to turn the crisis into an opportunity.

## Introduction

The COVID-19 crisis has been described as a *creeping mega-crisis* that was slow to arrive and slow to go away. It took most countries weeks, if not months, to return to a *new* normality characterised by face masks, physical distancing, and local lockdowns. The *creeping* nature of the pandemic presented new and complex challenges (Boin et al., 2020), and the COVID-19 crisis has extraordinary physical, social, and psychological impacts. Children’s lives were disrupted in many ways as efforts to contain the virus required the temporary closure of schools, childcare facilities, recreation centres, and many other amenities. Play was restricted in many ways, and children were physically separated from their friends and could not engage in most of the social activities they enjoy (Caffo et al., 2020; Fegert et al., 2020). As such, the COVID-19 pandemic, the resulting lockdowns and other containment measures may have converged into a highly stressful event for many children, dramatically changing their everyday lives.

The negative mental-health consequences of the COVID-19 pandemic on children have been documented. To date, several meta-analyses and systematic reviews of children’s mental health during the COVID-19 pandemic have indicated a high prevalence of depression (Nearchou et al., 2020; Ma et al., 2021; Panchal et al., 2021; Panda et al., 2021; Racine et al., 2021; Samji et al., 2021), anxiety (Nearchou et al., 2020; Ma et al., 2021; Panchal et al., 2021; Panda et al., 2021; Racine et al., 2021; Samji et al., 2021), irritability (Panchal et al., 2021; Panda et al., 2021), anger (Panchal et al., 2021), inattention (Panda et al., 2021), sleep disorders (Ma et al., 2021), and posttraumatic stress (PTSD; Ma et al., 2021). Current COVID-19 literature examines a number of risk factors that aggravate the impact of the pandemic on children’s mental health.

One pandemic-related variable that many studies look at is exposure. Research on previous disasters has shown that the severity of exposure to the disaster is strongly associated with the manifestation of mental health problems in children and that the development of posttraumatic stress symptoms in children exposed to a disaster depends on the degree of exposure (e.g., Lonigan et al., 1991; Korol et al., 2002). In the context of COVID-19, studies have also found that being infected oneself or having a relative who has been infected with or has died from COVID-19 are relevant risk factors for children’s mental health (e.g., Dönmez and Uçur, 2021; Luijten et al., 2021). Additionally, several qualitative and mixed-methods studies investigated COVID-19-related worries in children and adolescents during the early stages of the pandemic, when the lockdown was most severe. Given the medical profile and high mortality rate of COVID-19, concerns related to illness or death were to be expected. Some children lost their grandparents due to COVID-19. In addition, the widespread news of the deaths of elderly people (regardless of whether they were related to the children) may have triggered worrying thoughts about death and feelings of death anxiety (Sarkadi et al., 2021). In a German mixed-methods study, Vogel et al. (2021) found that in March 2020, 65.4% of the 9- to 19-year-old children and adolescents were worried about themselves, 97.0% about their families, and 82.2% about their friends. In a qualitative Italian study carried out in May-June 2020 (Segre et al., 2021), 75.6% of children aged 6–14 years expressed worries about their family members getting COVID-19. Similar results were obtained from an anonymous web survey carried out in Sweden in April–May 2020, containing both background and open-ended questions: worry about Corona was widespread among 4- to 18-year-old children and adolescents (77%) and was mostly related to disease or death (Sarkadi et al., 2021). In a quantitative study of 323 northern Italian primary school children in autumn 2020, Matiz et al. (2022) found that COVID-19 anxiety was positively correlated with negative affect. COVID-19 studies in adults show similar results, i.e., a high level of COVID-19-specific worry is associated with clinical levels of depression, anxiety, and posttraumatic stress (Fitzpatrick et al., 2020; Liu et al., 2020). In pre-COVID-research, the links between negative thoughts or worry and mental health problems have also been documented in children and adolescents (Stuijfzand et al., 2018).

In addition to the disaster-related (i.e., COVID-19-related) variables mentioned above, associations between sociodemographic variables and mental health outcomes are also known. Studies on children’s mental health during the COVID-19 pandemic often examine gender as an influencing factor, and meta-analyses consistently found that girls were more likely to experience negative mental health outcomes than boys, for example a higher prevalence of anxiety and depression (e.g., Fong and Iarocci, 2020; Ma et al., 2021; Racine et al., 2021). With regard to externalising problems, some studies show that boys exhibit more symptoms than girls (e.g., Giannotti et al., 2021; Schmidt et al., 2021). Location is also considered an important influencing factor, as infection and death rates differed, and countries used different strategies in their responses to the pandemic (Skinner et al., 2021). The studies came to contradictory results with regard to geographical area (rural vs. urban): Two studies with Chinese adolescents found a higher prevalence of anxiety and depressive symptoms in rural than in urban adolescents (Zhou et al., 2020; Xu et al., 2021), and Hine et al. (2020) argue that rural areas are particularly vulnerable in times of COVID-19. Conversely, Duan et al. (2020) found that residence in urban regions was associated with increased levels of anxiety among 7- to 18-year-old children. Sarkadi et al. (2021) found that geographical location (rural vs. urban) did not have an influence on the presence of worrying thoughts among children and adolescents in Sweden. Finally, when it comes to children’s mental health, socioeconomic variables are always relevant to consider (e.g., Reiss, 2013), especially in times of COVID-19 (e.g., Fong and Iarocci, 2020; Ravens-Sieberer et al., 2021) as some parents lost their jobs, suffered financial losses and faced challenges in securing food, housing, and other necessities for their children (Masten and Motti-Stefanidi, 2020).

In the aftermath of crises, stressful and traumatic events, psychology and psychiatry have traditionally had a *negative* focus. Their focus has been on the ways in which such experiences are precursors to highly distressing and sometimes severe psychological problems, maladaptive behaviours, and the course of disease (Tedeschi and Calhoun, 2004). Individuals will not necessarily develop psychiatric disorders, even in the face of the most traumatic circumstances, however, experiencing major crises usually leads to unpleasant psychological reactions and increases the risk of developing psychiatric problems (e.g., Rubonis and Bickman, 1991; Norris et al., 2002). There are also opportunities for positive change that result from taking on difficult circumstances, however, and a large number of studies have shown that individuals who have experienced traumatic events or crises also report positive changes (Tedeschi and Calhoun, 2004). Such positive changes have been conceptualised under the term posttraumatic growth (PTG), defined as the experience of positive change resulting from coping with very difficult life crises (Tedeschi and Calhoun, 1995). The five known dimensions of PTG are: (1) an increased appreciation of life in general, (2) more meaningful interpersonal relationships, (3) an increased sense of personal strength, (4) changed priorities, and (5) a richer existential and spiritual life (Tedeschi and Calhoun, 2004). The literature on PTG has typically focused on what Calhoun and Tedeschi (1998) termed *seismic psychological events*, i.e., events that challenge individuals’ assumptions about the society, the schematic structures that guide their sense of purpose, their plans for the future, and their understanding of the world. Ulset and von Soest (2022) noted that the COVID-19 pandemic may represent such a seismic psychological event for a significant number of young people, which may not only lead to significant distress but also promote PTG.

First studies in COVID-19 research using different subsamples have shown that in addition to various negative psychological reactions, some individuals reported PTG (e.g., health care workers: Feingold et al., 2022; parents/caregivers: Stallard et al., 2021; pregnant women: Chasson et al., 2022; therapists: Aafjes-van Doorn et al., 2022; U.S. military veterans: Na et al., 2021). However, despite the growing interest in PTG in the Corona crisis, few studies have examined the phenomenon in non-adults. Although some studies on PTG in adolescents during the COVID-19 pandemic have been conducted (Bhushan et al., 2022; Jian et al., 2022; Ulset and von Soest, 2022; Zhen and Zhou, 2022), research on children is still lacking. Zhen and Zhou (2022) found three heterogeneous patterns characterised by struggle (48.9%), distress (14.8%), and growth (36.3%) in 683 Chinese adolescents (15–18 years old). Another Chinese study of 2,090 adolescents (12–18 years old) found a PTG prevalence of 20.6%, and that the relationship between PTSD and PTG was an inverted U-shaped curve (Jian et al., 2022). In a representative survey of 12,686 junior and senior high school students from Oslo, Norway, Ulset and von Soest (2022), found that good mental health was only associated with higher degrees of PTG in groups that typically have a higher risk of adverse outcomes. In an Indian study, 68.9% of 9- to 20-year-old children and adolescents (only 3.7% of the participants were children, while the remaining 96.3% were adolescents) reported posttraumatic stress and 39.8% of those reporting posttraumatic stress were also experiencing PTG.

The main objective of this investigation was to examine the impact of the COVID-19 pandemic on children’s mental health (internalising problems, aggressive behaviour, posttraumatic stress symptoms) and health-related quality of life (HRQoL) during the first 2 years of the COVID-19 pandemic, i.e., at four measurement time points (t1 = March 2020, t2 = December 2020, t3 = June 2021; t4 = December 2021). Furthermore, the following research question on the precursors to children’s mental health burden in the COVID-19 pandemic was investigated: *Which COVID-19-related and sociodemographic variables are risk factors for children’s mental health during the COVID-19 pandemic?* To improve children’s mental health during the COVID-19 crisis, it is important to identify specific precursors of mental health problems. Based on previous studies, we expected pandemic exposure and threat experience, along with sociodemographic factors, to be fundamental risk factors for children’s mental health and HRQoL during the COVID-19 pandemic. We also explored positive changes in children from parents’ perspective, as it is known that people who face highly stressful events experience both negative and positive changes (Tedeschi and Calhoun, 2004). It is possible to experience constructive effects and the Corona crisis could also be a source of PTG (Bowling and Schumm, 2021). Based on this, and considering the novelty of the present crisis (e.g., compared to natural disasters or terrorist attacks), our second research question was: *What positive changes did parents observe in their children during the COVID-19 pandemic?*

## Materials and Methods

### Research Design

**FIGURE 1:**
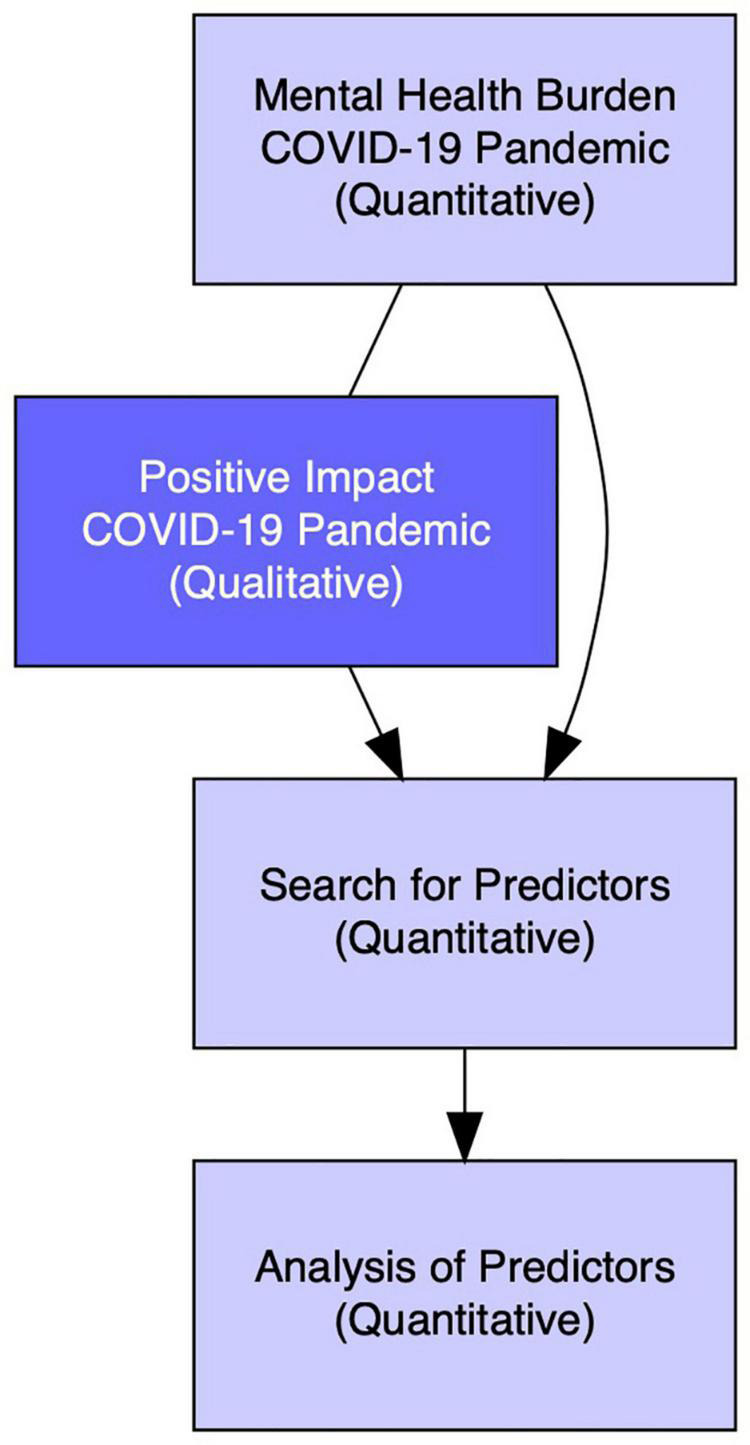
Concurrent quantitative and qualitative design.

In the present study a mixed-methods design was chosen: a *Concurrent Quan* + *Qual Design* according to Plano Clark and Ivankova (2016) which implies that the quantitative and qualitative research strands are conducted simultaneously. Our aim was for our qualitative findings to enhance our quantitative results. Negative mental health outcomes were studied quantitatively, positive changes qualitatively. In the subsequent search for predictors of mental health (quantitative analysis), both sociodemographic and COVID-19 related risk factors, as well as positive changes (the open-ended question was dichotomised as perceived PTG), were considered. Figure 1 shows how quantitative and qualitative approaches were combined in this study.

### Setting and Procedure

This study is part of the *Tyrolean COVID-19 Children’s Study*, a large mixed-methods research project funded by the *Land Tyrol* and conducted by the *Department of Child and Adolescent Psychiatry Innsbruck* (Austria). It focuses on the impact of the COVID-19 pandemic on 3- to 13-year-old Tyrolean children, from the perspectives of children, parents, and educators. In this sub-study only data from parents have been examined. North Tyrol (Austria) and South Tyrol (Italy), two regions located in the Alps, were among the European regions most affected by COVID-19 in the initial phase of the pandemic, as the Paznaun Valley in North Tyrol and the Gardena Valley in South Tyrol – two well-known winter tourism destinations – had become high-risk areas, i.e., Corona hotspots, in February 2020 (Land Tirol, 2020a; Melotti et al., 2021). Although North and South Tyrol belong to two different states, they have close historical and geographical links and, together with the Autonomous Province of Trento, form the cross-border European region of *Tyrol – South Tyrol – Trentino* (Choffy, 1961; Raffeiner, 2019).

This study was an online-study and was conducted using the software *LimeSurvey* (LimeSurvey GmbH, 2021) for the first measurement time point (t1), and *CHES* (ESD, 2020) for the following measurement time points. The study consists of four assessment waves (t1 = March/April 2020, t2 = December 2020/January 2021, t3 = June/July 2021, t4 = December 2021/January 2022).

Every school in the Tyrolean Corona hotspots joined the study at t1. The North Tyrolean Corona hotspots were the Paznaun Valley, St. Anton, and Sölden (Land Tirol, 2020a,b), and the Gardena Valley was the South Tyrolean Corona hotspot (Melotti et al., 2021). Five other South Tyrolean municipalities (Castelrotto, Braies, Villabassa, Corvara, and La Valle) were also defined as Corona hotspots, i.e., municipalities with the highest ratio of people testing positive (Autonome Provinz Bozen – Südtirol, 2020) to inhabitants (Autonomous Province of South Tyrol – Provincial Statistics Institute, 2020) at the end of May 2020. In June 2020, schools in the Corona hotspots asked parents to retrospectively report on children’s mental health during the first lockdown in March 2020. For subsequent measurement time points (t2, t3, t4), parents were recruited through the schools that had participated in t1 and reported on the current state of their children’s mental health at each time point. From measurement time point 2 onward the study was also advertised through North and South Tyrolian media (print media as well as online formats) to encourage broad participation.

The parents gave their written informed consent. They could withdraw from further participation at any stage of the survey without needing to give a reason. If any psychological problems or questions arose, the parents could contact the outpatient clinic or the last author of this study. The study was approved by the ethics committee of the *Medical University of Innsbruck* (No.: 1183/2020). The ethical vote of the *Medical University of Innsbruck* also applies to the German-speaking population of South Tyrol. This was clarified verbally with the president of the *South Tyrolean Ethics Committee*.

### Participants

A total of *N* = 2,691 questionnaires were filled out by 90.9% mothers and 7.9% fathers (1.2% not specified) who provided information on their 3- to 13-year-old children (48.8% daughters). The study consisted of four assessment waves: 1,972 parents participated once, 312 two or more times. Of the 2,691 cases, 951 were grouped as younger children, i.e., preschool children (between 3 and 6 years old, *M*_*age*_ = 4.7, *SD*_*age*_ = 1.1, 47.1% girls) and 1,740 were grouped as older children, i.e., schoolchildren (between 7 and 13 years old, *M*_*age*_ = 9.6, *SD*_*age*_ = 1.9, 49.8% girls). A total of 2,652 parents answered the open-ended question (937 younger age group; 1,715 older age group). Further characteristics of the sample, including children’s sociodemographic and COVID-19-related variables are shown in Table 1. Eligibility criteria were the place of residence (North or South Tyrol), parenthood of a 3- to 13-year-old child, proficiency in the German language, and the cognitive ability to fill out an online questionnaire. Our sample was opportunistic and not representative, as only parents from the Tyrolean Corona hotspots or parents who had access to the study link through media advertising participated in the online survey.

**TABLE 1:** Sociodemographic data.

		*n*	%
Total sample size		2691	100
**Measurement time points**			
	March 2020 (t1)	436	16.2
	December 2020 (t2)	649	24.1
	June 2021 (t3)	505	18.8
	December 2021 (t4)	1101	40.9
**Age**			
	Younger children (3–6 years)	951	35.3
	Older children (7–13 years)	1740	64.7
**Gender**			
	Girl	1314	48.8
	Boy	1377	51.2
**Region**			
	North Tyrol	1648	61.2
	South Tyrol	1043	38.8
**Geographical location**			
	Rural	2216	82.3
	Urban	475	17.7
**Economic disruption**			
	Yes	456	16.9
	No	2235	83.1
**Pandemic exposure**			
	Child Had COVID-19	354	13.2
	Family Member Had COVID-19	1153	42.8
	Family Member Hospitalized for COVID-19	192	7.1
	Family Member Died from COVID-19	90	3.3
**Threat experience**			
	COVID-19-Infection	782	31.7
	COVID-19-Infection of Family Member	1256	51.2
	Death from COVID-19	250	10
	Death of Family Member from COVID-19	803	33.3

*Data for March 2020 (t1) were collected retrospectively in June 2020.*

### Measures

The online survey was provided in German, since the study was conducted in German speaking areas (North Tyrol in Austria and South Tyrol in Italy). The following standardised and validated German measures were used. The wording of the instructions was adapted to the Corona situation.

#### Demographic Characteristics

Parents indicated their own gender and nationality (North Tyrol, Austria/South Tyrol, Italy), geographical location (rural/urban; urban was defined as *municipalities with more than 15,000 inhabitants*) and experience of economic disruption related to the Corona crisis (“Have you and your family experienced financial problems due to the Corona crisis?”). The child’s age and gender were also reported.

#### Open-Ended Question After the Standardised Questionnaires: Potentially Positive Effects of the COVID-19 Pandemic

Parents were asked to describe potentially positive effects of the Corona crisis on their children (“What positive impact do you think the Corona crisis has/had on your child?”).

#### Pandemic Exposure

Parents provided information on their child’s degree of exposure through four yes/no questions: the child themselves had COVID-19; a family member had COVID-19; a family member was hospitalised with COVID-19; a family member died from COVID-19.

#### Threat Experience

Parents provided information about the threat of coronavirus experienced by their child through four yes/no questions: worry that a family member could become ill; worry that the child could themselves become ill; worry that a family member could die; worry that the child themselves could die.

#### Child and Adolescent Trauma Screen – Caregiver Report (CATS-C-D 3-6/CATS-C-D 7-17, German Version)

The *CATS-C-D* (Berliner and Goldbeck, 2014a,b) screens the child’s risk of PTSD from the caregiver’s perspective [e.g., “Having very negative emotional states (afraid, angry, guilty, ashamed)”; *CATS-C-D 3-6*: 16 items; *CATS-C-D 7-17*: 20 items]. Because it is based on the *DSM-5* criteria, it measures symptoms of the following four dimensions: re-experiencing, avoidance, negative mood-cognitions, and arousal. The items are rated on a 4-point Likert scale that ranges from 0 (*never*) to 3 (*almost always*). The total score is calculated by summing the items (scale range: 0–60) (Ulmer Onlineklinik, n.d.). A Cronbach’s alpha of α = 0.88 for the full scale was found in a German sample of 95 reports by caregivers of 7- to 17-year-old children and adolescents (Sachser et al., 2017). In the present study, the internal consistency for the full scale was good for the younger (α = 0.89) and excellent for the older children (α = 0.92).

#### Child Behaviour Checklist (CBCL 1,5-5/CBCL 6-18R, German Version)

By completing the CBCL (Achenbach and Arbeitsgruppe Deutsche Child Behavior Checklist, 2000; Döpfner et al., 2014), parents rate their child’s behavioural problems, emotional problems, somatic complaints, and social skills on a 3-point Likert scale ranging from 0 (*not true*) to 2 (*very true or often true*) (Achenbach and Arbeitsgruppe Deutsche Child Behavior Checklist, 2000; Döpfner et al., 2014). Cronbach’s alphas of α = 0.73 and α = 0.80, for girls and boys respectively, were found in a field sample of 985 schoolchildren (6–11 years) (Walter and Remschmidt, 1999). This study used the problem scale *Internalising Problems* and the subscale *Aggressive Behaviour* of the *CBCL*.

##### Problem Scale “Internalising Problems”

The problem scale *Internalising Problems* comprises problems that are mainly within the self (e.g., “Too fearful or anxious”). For the younger children it consists of four subscales (*Emotionally Reactive* – 8 items, *Anxious/Depressed –* 8 items, *Somatic Complaints* – 11 items, *Withdrawn/Depressed* – 8 items; Achenbach and Arbeitsgruppe Deutsche Child Behavior Checklist, 2000), for the older children of three (*Anxious/Depressed* – 13 items, *Withdrawn/Depressed* – 8 items, *Somatic Complaints* – 11 items; Döpfner et al., 2014). In the present study, the internal consistency for the problem scale *Internalising Problems* was excellent for both the younger (α = 0.91) and the older children (α = 0.92).

##### Subscale “Aggressive Behaviour”

The subscale *Aggressive Behaviour* is computed by summing 20 items (scale range: 0–40) for the younger children (Achenbach and Arbeitsgruppe Deutsche Child Behavior Checklist, 2000) and 18 items (scale range: 0–36) for the older children (e.g., “Easily frustrated.”). Aggressive behaviour is attributed to external behaviour (Döpfner et al., 2014). In the present study, the internal consistency for the subscale *Aggressive Behaviour* was excellent both for the younger (α = 0.91) and the older children (α = 0.91).

#### Kiddy-KINDL^R^/KINDL^R^ – Parents’ Version (Kiddy-KINDL^R^ 3-6/KINDL^R^ 7-13)

The Kiddy-KINDL^R^/KINDL^R^ (Ravens-Sieberer and Bullinger, 2000a,b) is a 24-item parent-report about the child’s HRQoL (e.g., “My child felt alone.”). The HRQoL is assessed by means of six subscales: physical and emotional well-being, self-esteem, family, friends, (pre)school. Each subscale consists of 4 items presented on a 5-point Likert scale that ranges from 1 (*never*) to 5 (*always*). When analysing the *Kiddy-KINDL^R^*/*KINDL*^R^, the subscale scores are converted to a range of 0–100 (Ravens-Sieberer and Bullinger, 2000c). In a German sample (3,875 3- to 6-year-old children; 4,148 7- to 10-year-old children) Cronbach’s alphas of α = 0.82 (younger children) and α = 0.82 (older children) were found for the full scale (Ravens-Sieberer et al., 2007). In the present study, the internal consistency for the full scale was good for the younger (α = 0.85) and excellent for the older children (α = 0.91).

### Data Analysis

#### Quantitative Data Analysis

To determine differences in mental health outcomes over the course of the pandemic, the absolute and relative frequencies of the categorical HRQoL and mental health outcomes were first calculated for each measurement time point (t1, t2, t3, and t4). For this, *CBCL* raw scores were converted into *T*-scores. *T*-scores were classified as *normal* (*Internalising Problems:* < 60; *Aggressive Behaviour:* < 65), *borderline* (*Internalising Problems:* 60–63; *Aggressive Behaviour:* 65–69), and *clinical* (*Internalising Problems:*≥64; *Aggressive Behaviour:* ≥70) (Achenbach and Rescorla, 2000; Döpfner et al., 2014). *CATS* raw scores were classified as *normal* (3–6 years: 0–11; 7–13 years: 0–14), *moderate distress* (3–6 years: 12–14; 7–13 years: 12–14), and *probable PTSD* (3–6 years: 15+; 7–13 years: 21+) (Ulmer Onlineklinik, n.d.). Secondly, to test the differences in clinical classifications (normal vs. clinical) between t1 and t4 and between preschool and schoolchildren at t4, inductive statistics were measured to obtain *p*-values (Fisher’s exact test) and effect sizes Φ (phi coefficient).

The open-ended question (“What positive impact do you think the Corona crisis has/had on your child?”) was dichotomised as *perceived PTG* for quantitative analysis. If parents noticed one or more positive changes, the quantitative predictor variable *perceived PTG* was a 1, if parents stated that they did not notice any positive changes, *perceived PTG* was a 0.

To identify risk factors, mixed model panel regression analyses were conducted. Sociodemographic variables and COVID-19-related variables were included as fixed effects. The simultaneous inclusion of these predictors in the multilevel models allowed for thorough control of confounding factors. As some parents participated more than once in the survey, a random intercept for each participant was included (to account for and represent individual baselines). Since time-independent predictors were sought, the time of measurement was also included in the model as a random effect. Originally, a separate multilevel model was created for each outcome for both age groups (3–6 and 7–13 years). After it was shown that the associations between the predictors and the outcomes did not differ between the two age groups, the models were combined and only one model was created for each outcome, using the age group as an additional random effect. Perceived PTG was the only predictor moderated by the age group. Therefore, a perceived PTG slope was calculated for each age group by including an interaction between perceived PTG and age group in the model. Multilevel modelling was conducted with R version 4.1 and the lmerTest package. Standardised regression coefficients (β) for each predictor were calculated for each outcome by multiplying the unstandardised regression coefficient by the standard deviation of the predictor and dividing by the standard deviation of the outcome. β corresponds to the effect size (Lorah, 2018) of a predictor on the outcome. The 95% confidence levels were estimated using standard errors of the unstandardised regression coefficients. The intraclass correlation coefficient (ICC) was calculated for each random effect. Marginal and conditional *R*^2^ are reported as a summary for the multilevel model fit (Nakagawa and Schielzeth, 2013).

Differences between the measurement time points in the predictors threat experience, economic disruption, and perceived PTG were determined separately for each age group between March 2020 (t1) and December 2021 (t4). A regression model was constructed for each of these three predictors with gender, region, and urban living as covariates. The same was done for the direct comparison of the two age groups at t4. The standardised regression coefficients (β) of the regression analysis were reported as effect sizes.

*P*-values ≤ 5% were considered significant. When effects of predictors were reported, a Bonferroni correction to the significance level was applied to keep the family-wise error rate at approximately 5%. Effect sizes (Φ, β and ICC) < 0.1 were considered negligible, ≥0.1 small, ≥0.3 medium, and ≥0.5 large.

#### Qualitative Data Analysis

Qualitative data were analysed with MAXQDA 2020 software for qualitative data analysis, using Thematic Analysis (TA; Braun and Clarke, 2006). The qualitative data analysis was conducted primarily by the last author in collaboration with the first author. We used an inductive approach to TA, i.e., codes and themes were directed by the data content. Our aim was to understand the possible positive effects of the Corona crisis on children from parents’ perspective. This goal was achieved through the following steps of the TA (Braun and Clarke, 2006): (1) in order to develop and generate initial ideas about the data, first and last authors familiarised themselves with the written responses by reading and rereading the texts. (2) The last author then began the process of coding and analysis by focusing on the semantic content of the participants’ written responses. (3) Where the semantic content was not obvious at first glance, the last author consulted the first author and the two discussed until a consensus was reached. (4) The codes were clustered to generate themes. (5) Finally, these themes were grouped and regrouped to enable us to review the themes. (6) In conclusion, we developed codes to explicate the range of themes.

In order to compare the younger and the older age group with regard to the identified themes, we conducted a cross-tabulation within the mixed-methods section of MAXQDA. Cross-tabulation enabled us to compare the frequencies of the codes for the groupings (younger age group vs. older age group).

## Results

### Children’s Mental Health Burden During the COVID-19 Pandemic (Quantitative)

**FIGURE 2:**
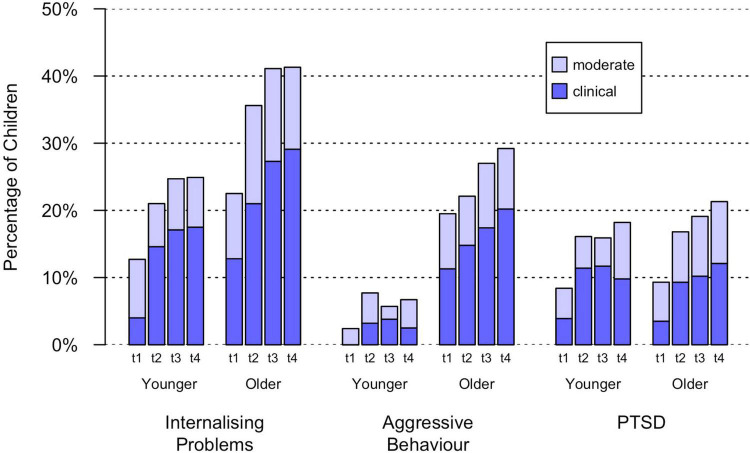
Percentage of children with clinical relevant classifications of internalising problems, aggressive behaviour and posttraumatic stress symptoms. Younger: parent-report on 3–6-year-old children; Older: parent-report on 7–13-year-old children; t1 = March 2020; t2 = December 2020; t3 = June 2021; t4 = December 2021.

With regard to the younger age group (preschool children), the number of children with a clinical classification of internalising problems was higher at t4 in December 2021 than at t1 in March 2020 (*n*_t1_ = 4.0%, *n*_t4_ = 17.5%, Φ = 0.17, *p* < 0.001). This was also true for the older children, i.e., schoolchildren (*n*_t1_ = 12.8%, *n*_t4_ = 29.1%, Φ = 0.19, *p* < 0.001). A comparison of the two age groups in December 2021 showed that more schoolchildren than preschool children had a clinical classification of internalising problems (Φ = 0.16, *p* < 0.001). The number of preschool children with clinical manifestations of aggressive behaviour was both low in March 2020 as well as in December 2021 (*n*_t1_ = 0.0%, *n*_t4_ = 2.5%, Φ = 0.08, *p* = 0.128). Conversely, the number of schoolchildren showing aggressive behaviour was higher in December 2021 than in March 2020 (*n*_t1_ = 11.3%, *n*_t4_ = 20.2%, Φ = 0.11, *p* = 0.001). A comparison of the two age groups in December 2021 showed that the proportion of schoolchildren with a clinical classification of aggressive behaviour was greater than that of preschool children (Φ = 0.27, *p* = 0.001). The number of younger children with clinically relevant posttraumatic stress symptoms was higher in December 2021 than in March 2020 (*n*_t1_ = 3.9%, *n*_t4_ = 9.8%, Φ = 0.11, *p* = 0.010). This was also true for the schoolchildren (*n*_t1_ = 3.5%, *n*_t4_ = 12.1%, Φ = 0.14, *p* < 0.001). In December 2021, there was no difference between the two age groups in terms of the proportion of children with probable PTSD (Φ = 0.04, *p* = 0.238). All reported effects were small. The relative number of children in each classification group for each measurement point can be seen in Figure 2.

### Posttraumatic Growth in Children During the COVID-19 Pandemic (Qualitative)

The final themes identified with TA were “importance of intra- and extra-familial relationships” (Theme 1), “new competences and experiences” (Theme 2), “values and virtues” (Theme 3), “use of time” (Theme 4), and “family strength” (Theme 5). It is clear that the most significant event during the Corona crisis was the lockdown with accompanying quarantine in March 2020, as even though the question about possible positive changes was asked separately at each of the four measurement time points all of the changes in children as observed by their parents related to this point in time. Since similar statements were made across all measurement time points, it can be assumed that these changes are stable. This means that the identified themes are consistent with the factors of PTG. Table 2 shows the extent to which these themes affected the younger vs. older age group. It was particularly noticeable within the group of younger children, that parents and toddlers enjoyed spending time together very much. The bonds between children and their fathers and siblings seemed especially strengthened. In general, the families of both age groups became more cohesive, and a deceleration in everyday life was observed. For the older children, the Corona crisis mainly promoted the development of independence and the acquisition of new skills and capabilities. In this age group, an increase in basic moral values was observed, too. In the following, we present the five themes one by one with illustrative data examples.

**TABLE 2:** Perceived posttraumatic growth in children during the COVID-19 pandemic.

	Preschool children (≤6 years)	School children (>6 years)	Total
**Theme 1: Importance of intra- and extra-familial relationships** **(corresponding to PTG factor “relating to others”)**	*CPSTABLEENTER* **73.6%**	*CPSTABLEENTER* **43.3%**	53.2%
Growing together as a family, cohesion, joint actions (+)	15.1%	15.6%	15.4%
A lot of time with parents and family	**39.1%**	**21.6%**	27.8%
Dad and relationship with sibling(s) (+)	**16.9%**	**6.2%**	10.0%
Extended relationships	2.5%	1.0%	1.5%
**Theme 2: New competences and experiences** **(corresponding to PTG factor “new possibilities”)**	*CPSTABLEENTER* **7.5%**	*CPSTABLEENTER* **30.9%**	22.6%
Independence, adaptability	**3.6%**	**17.8%**	12.7%
New experiences incl. home schooling	3.1%	6.6%	5.4%
Digitalisation, new competences	**0.8%**	**6.5%**	4.5%
**Theme 3: Values and virtues** **(corresponding to PTG factor “appreciation of life”)**	*CPSTABLEENTER* **7.4%**	*CPSTABLEENTER* **11.9%**	10.3%
Appreciation, virtues, doing something good for others (+)	**6.6%**	**11.1%**	9.5%
Mindfulness, taking care of yourself	0.8%	0.8%	0.8%
**Theme 4: Use of time** **(corresponding to PTG factor “appreciation of life”)**	*CPSTABLEENTER*10.6%	*CPSTABLEENTER*10.0%	10.3%
Creativity, fantasy, time	2.4%	1.7%	2.0%
Deceleration	8.2%	8.3%	8.3%
**Theme 5: Family strength** **(corresponding to PTG factor “personal strengths”)**	*CPSTABLEENTER*0.9%	*CPSTABLEENTER*2.7%	2.1%
Sum	100.0%	100.0%	100.0%
*N* = documents	937	1,715	2,652

*Percentages in bold indicate significant differences between the two age groups.*

#### Theme 1: Importance of Intra- and Extra-Familial Relationships (Corresponding to Posttraumatic Growth Factor “Relating to Others”)

The most prominent positive theme resulting from the Corona crisis was intra-familial relationships. This means that both quantitative (the time children and caregivers spend together) and qualitative aspects (e.g., family cohesion) of parent–child relationships were of great importance. For example, one mother said: “I spent more time with them doing crafts, painting, listening. I have consciously spent more time with my children.” Family cohesion was described as follows*:* “We grew even closer as a family, in the sense that we had ‘only’ each other.” Relationships within the family intensified, i.e., due to reduced work hours or the requirement to work from home, fathers also spent more time at home and thus spent more time with the child. Relationships with siblings intensified as contact with people outside the household was not allowed: “She built up a very intimate and friendly relationship with her sister. I remember the sentence: ‘I didn’t know you could play so well with L.”’

Extra-familial contacts mainly concern closer relationships with (distant) relatives as well as relationships with children with whom the child was not friends before the crisis but who were brought together by chance. For example, parents reported that their children made new friends despite severe contact restrictions. This occurred because school classes were divided in order to reduce the number of children in each classroom. Therefore, children got to know classmates better and became friends: “New friendships through shift work [school classes were halved to reduce the risk of contagion; author’s note] in the first lockdown.”

#### Theme 2: New Competences and Experiences (Corresponding to Posttraumatic Growth Factor “New Possibilities”)

Parents reported that the challenges of the Corona crisis brought about many changes to their children’s development and experiences. Emphasis was placed on the independence they acquired over time, mainly related to the independent learning of school subject material: “Homeschooling has taught the child to do tasks more independently – but this only worked out in the second lockdown. Complete disaster during the first lockdown.” In general, this increased independence has strengthened the children’s self-confidence. Whilst being homeschooled (online lessons), the children also acquired additional digital skills: “Homeschooling enabled them to expand their digital skills.” The children also began to apply these newly acquired digital skills more and more to their personal lives: “They also meet friends online.” Parents reported that children had new experiences, especially in their *life at home*. As one mother put it: “The child sees what the daily life of the other family members is like and is not at school for most of the day.” Homeschooling was seen in a somewhat positive light, as it allowed for more flexibility and removed the stress of the daily morning routine: getting up, getting dressed etc.

#### Theme 3: Values and Virtues (Corresponding to Posttraumatic Growth Factor “Appreciation of Life”)

Basic human values such as consideration for others, benevolence, etc. came to the fore again during the containment measures to help keep the propagation of the virus under control. The specific containment measures such as hygiene measures, school closures, physical distancing, movement restriction, and working from home, as well as the temporary closures of non-essential businesses drastically restricted people’s lives. People’s social lives were conducted mainly at home and the family members had to find ways to live together harmoniously. Parents acknowledged children’s efforts to create a harmonious family life and noted the following behaviours and/or virtues in their children: “Everyone had to learn to be considerate of each other.”; “Better understanding when needs cannot be met immediately (e.g., going to a certain swimming pool, restaurant, etc.)”; “Understanding that one can accept restrictions out of respect for others.” After a total of four lockdowns in Austria, the parents noticed that the children took things less for granted. They learned to appreciate the value of everyday things and actions: “More awareness of what it means to have friends and to be allowed to go to school.” or “Less time pressure due to less leisure programme.” Most parents spoke of deceleration in everyday life and they recognised that both they and their children lead stressful lives.

#### Theme 4: Use of Time (Corresponding to Posttraumatic Growth Factor “Appreciation of Life”)

Thrown completely back on themselves and the family by the containment measures, children and parents alike had to learn how to deal with their newly acquired time. Having time, taking time for something, finding time for oneself, and using time differently were the most prominent topics of theme 4. Many parents appreciated having more time for their family and for joint activities. Before Corona, leisure activities – as enjoyable as they were – were often the cause of stress: “More time at home and more ‘free time,’ because all other activities (sports, courses etc.) have been dropped and also otherwise less social commitments (also at weekends). Kind of more peace and slowness in everything.” Parents noted that the increase in free time promoted children’s creativity and stimulated their imagination.

#### Theme 5: Family Strength (Corresponding to Posttraumatic Growth Factor “Personal Strength”)

Family strength relates mainly to children developing an understanding that the unexpected can happen and that people must learn to adapt and manage it. Parents found it important that their children see that crises can be overcome: “He understood that negative things can happen. We can find a solution.” Parents also emphasised the significance of overcoming a crisis *together*. Children should learn from the crisis and look at both sides of the coin – the crisis brings positive as well as negative things: “Knowing that she has the ability to always look at everything anew and find something good.” This theme also relates to understanding a *bigger picture* and understanding that everyone is a part of it and should care about “experiencing the fragility and unpredictability of human existence and understanding the value of life and health.”

### Predictors of Mental Health

**FIGURE 3:**
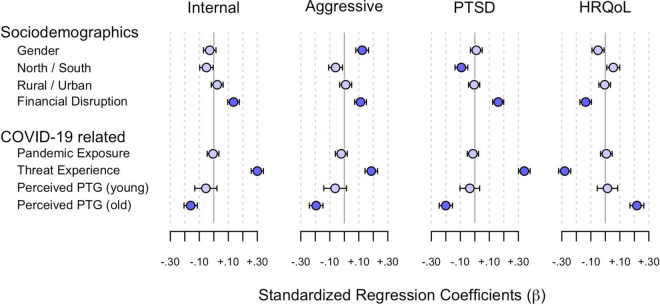
Predictor effects on internalising problems, aggressive behaviour, posttraumatic stress symptoms and HRQoL. Standardized regression coefficients (β) mean and 95% confidence intervals are shown. Significant effect sizes are in dark blue.

The results of multilevel modelling, i.e. time- and age-independent predictors of mental health outcomes (internalising problems, aggressive behaviour, posttraumatic stress symptoms, and HRQoL; see Table 3), are presented below. Threat experience and economic disruption led to an increase in internalising problems, aggressive behaviour, and posttraumatic stress symptoms as well as a lower HRQoL, whilst perceived PTG led to a decrease in internalising problems, aggressive behaviour and posttraumatic symptoms as well as increased HRQoL in older children. Boys showed more aggressive behaviour than girls. North Tyrolean children showed more posttraumatic stress symptoms, but this effect is negligible because of the size of the effect. The effect sizes are shown in Figure 3. In the following, all of the results for internalising problems, aggressive behaviour, posttraumatic stress symptoms, and HRQoL are presented in detail.

**TABLE 3:** Mental health scores as a function of socioeconomic and COVID-19-related variables.

	Internal		Aggressive		PTSD		HRQoL	
	B	*SE*	B	*SE*	B	*SE*	B	*SE*
**Regression coefficients (fixed effects)**								
Intercept	7.82	0.72	7.13	0.71	5.77	1.49	75.65	2.65
Gender	–0.48	0.38	1.59*	0.29	0.16	0.33	–1.36	0.57
North/South	–0.88	0.42	–0.78	0.32	–1.50*	0.36	1.53	0.63
Rural/Urban	0.53	0.47	0.17	0.36	–0.10	0.41	–0.17	0.72
Economic Disruption	3.12*	0.47	1.92*	0.36	3.37*	0.41	–4.86*	0.73
Pandemic Exposure	–0.20	0.84	–0.62	0.64	–0.48	0.73	0.44	1.30
Threat Experience	7.03*	0.50	3.22*	0.38	7.23*	0.44	–10.15*	0.78
Perceived PTG (young)	–0.98	0.52	–0.58	0.54	–0.78	0.62	0.72	1.11
Perceived PTG (old)	–3.25*	0.43	–3.04*	0.35	–3.59*	0.42	6.69*	0.76
**Variance components (random effects)**				
Time of meas. ICC	0.04	0.04	0.03	0.01
Participants ICC	0.67	0.66	0.59	0.50
Age Group ICC	0.00	0.03	0.15	0.14
**Model statistics**				
$R_{\mathrm{GLMM}\,\left(m\right)}^{2}$	0.15	0.11	0.19	0.15
$R_{\mathrm{GLMM}\,\left(c\right)}^{2}$	0.72	0.70	0.70	0.61

*p ≤ 0.05; Bonferroni significance level correction for 28 comparisons; Gender: 0 = female, 1 = male; North/South: 0 = North Tyrol, 1 = South Tyrol; Rural/Urban: 0 = Rural, 1 = Urban; Perceived PTG: 0 = no perceived PTG, 1 = perceived PTG; ICC = intraclass correlation coefficient; $R_{G\,L\,M\,M\,\left(m\right)}^{2}=m\,a\,r\,g\,i\,n\,a\,l\,R^{2}$; $R_{G\,L\,M\,M\,\left(c\right)}^{2}=c\,o\,n\,d\,i\,t\,i\,o\,n\,a\,l\,R^{2}$.

#### Internalising Problems

Internalising problems increased with economic disruption (β = 0.14, *p* < 0.001) and threat experience (β = 0.30, *p* < 0.001). The effect was small for economic disruption and medium for threat experience. Gender (β = –0.03, *p* = 0.202), region (β = –0.05, *p* = 0.036), living in a town/city (β = 0.02, *p* = 0.257), and pandemic exposure to COVID-19 (β = –0.01, *p* = 0.810) did not influence internalising problems. Perceived PTG reduced internalising problems in older children (β = –0.16, *p* < 0.001), but not in younger ones (β = –0.05, *p* = 0.063). This effect was small.

#### Aggressive Behaviour

Boys were more likely to show aggressive behaviour than girls (β = 0.12, *p* < 0.001). Economic disruption (β = 0.11, *p* < 0.001) and threat experience (β = 0.19, *p* < 0.001) also led to more aggressive behaviour. Region (β = –0.06, *p* = 0.014), urban living (β = 0.01, *p* = 0.640) and pandemic exposure to COVID-19 (β = –0.02, *p* = 0.331) had no influence. Perceived PTG reduced aggressive behaviour in older children (β = –0.19, *p* < 0.001), but not in younger ones (β = –0.06, *p* = 0.286). All effects were small.

#### Posttraumatic Stress Symptoms

Posttraumatic stress symptoms increased when the family experienced economic disruption (β = 0.16, *p* < 0.001) or children experienced more threat (β = 0.34, *p* < 0.001). The effect was small for economic disruption and medium for threat experience. North Tyrolean children were more likely to experience posttraumatic stress symptoms than South Tyrolean children, but this effect was negligible (β = –0.09, *p* < 0.001). Gender (β = 0.01, *p* = 0.620), pandemic exposure to COVID-19 (β = –0.01, *p* = 0.508) and urban living (β = 0.00, *p* = 0.808) showed no influence. Perceived PTG reduced posttraumatic stress symptoms in older children (β = –0.20, *p* < 0.001), but not in younger ones (β = –0.03, *p* = 0.213). This effect was small.

#### Health-Related Quality of Life

Health-related quality of life declined with economic disruption (β = –0.14, *p* < 0.001) and threat experience (β = –0.28, *p* < 0.001). In contrast, gender (β = –0.05, *p* = 0.017), region (β = 0.05, *p* = 0.016), living in a town/city (β = 0.00, *p* = 0.815) and pandemic exposure to COVID-19 (β = 0.01, *p* = 0.733) had no influence. Perceived PTG increased HRQoL in older children (β = –0.22, *p* < 0.001), but not in younger ones (β = 0.01, *p* = 0.518). All effects were small.

### Significant Predictors in Detail

**FIGURE 4:**
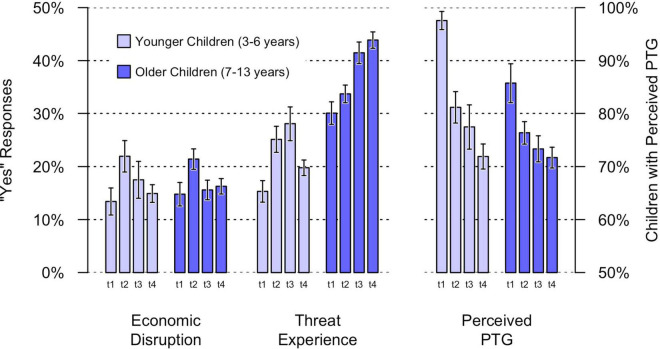
Predictor scores of economic disruption, threat experience and perceived PTG. Error bars are standard errors of the mean; predictor scores are the arithmetic mean of four items for threat experience and pandemic exposure and single item score for economic disruption and perceived PTG; t1 = March 2020; t2 = December 2020; t3 = June 2021; t4 = December 2021.

Scale scores for economic disruption, pandemic exposure, threat experience and perceived PTG for the single measurement points can be seen in Figure 4. Results are presented in detail, i.e., over time (t1 = March 2020 vs. t4 = December 2021) and for both age groups (younger = preschool children vs. older = schoolchildren).

#### Economic Disruption

There was no difference in economic disruption between March 2020 (t1) and December 2021 (t4), in either the families with younger children (β = 0.02, *p* = 0.667) or the families with older children (β = 0.00, *p* = 0.948). A comparison of the two age groups in December 2021 (t4) showed that they did not differ (β = 0.02, *p* = 0.596). Among younger children, in March 2020 (t1) 13.4% of parents (14.8% among older children) reported that the family had experienced financial problems due to the Corona crisis. In December 2021 (t4), 14.9% of parents of younger children (16.3% for older children) reported economic disruption due to the Corona crisis.

#### Threat Experience

Younger children’s threat experience was not elevated in December 2021 (t4) compared to t1 in March 2020 (β = 0.05, *p* = 0.180), but older children’s was (β = 0.12, *p* < 0.001). In December 2021 (t4), older children had a higher level of threat experience than younger children (β = 0.29, *p* < 0.001): 63.7% of parents of older children (52.5% in March 2020) answered yes to at least one of the four questions about threat experience. For younger children, this was only 34.4% (29.6% in March 2020).

#### Perceived Posttraumatic Growth

Parents in December 2021 (t4) reported less PTG for younger children (β = –0.22, *p* < 0.001) and older children (β = –0.11, *p* = 0.009) than parents in March 2020 (t1). PTG in December 2021 (t4) did not differ between younger and older children (β = –0.01, *p* = 0.763). Though almost every child (97.6% of younger, 85.7% of older children) showed signs of PTG in March 2020 (t1), almost three quarters of children (71.9% of younger, 71.7% of older children) showed signs of PTG in December 2021 (t4).

## Discussion

The aim of this mixed-methods study was to investigate the impact of the first 2 years of the COVID-19 pandemic on the mental health and HRQoL of Tyrolean preschool children and schoolchildren. Children’s mental health outcomes (internalising problems, posttraumatic stress symptoms) in both age groups were worse in December 2021 (t4) than children’s mental health outcomes in March 2020 (t1). A difference in aggressive behaviour was only found among schoolchildren. The parents also reported the following positive changes in their children during the Corona crisis: (1) the importance of intra- and extra-familial relationships, (2) new competences and experiences, (3) values and virtues, (4) use of time, and (5) family strength. Additionally, this study examined how the impact of the pandemic differed depending on specific sociodemographic and COVID-19-related variables. In multilevel modelling, threat experience, economic disruption, and perceived PTG were shown to be the strongest predictors of mental health outcomes and HRQoL. In addition, male gender was shown to be a predictor of aggressive behaviour. In terms of age, schoolchildren showed more internalising problems, aggressive behaviour, and threat experience than preschool children. With regard to time (t1 = March 2020 vs. t4 = December 2021), parents at t4 reported more threat experience in older children and less PTG in both older and younger children than parents at the beginning of the pandemic, while there was no difference in economic disruption.

Our findings are consistent with other COVID-19 results in that we found a high prevalence of clinically relevant symptoms (Ma et al., 2021) and symptoms became more pronounced as the Corona crisis progressed. However, our results are of cross-sectional nature. Indeed, COVID-19 studies with children have been almost exclusively cross-sectional and longitudinal data is still scarce (Wade et al., 2020). Waite et al. (2021) found a worsening of mental health symptoms in United Kingdom preadolescent children from April to May 2020: a 10% increase in those who met the probable case criteria for emotional symptoms, a 20% increase in hyperactivity/inattention, and a 35% increase in conduct problems. A Canadian study (Hawke et al., 2021) showed that for the majority of adolescents, mood concerns and COVID-19-related worries increased early in the pandemic, declined over the summer months and subsequently increased again in autumn 2020. In the representative German study by Ravens-Sieberer et al. (2022), the incidence of general mental health problems, anxiety and depressive symptoms in children and adolescents increased during the first two waves (wave 1: May/June 2020, wave 2: December 2020/January 2021), followed by a slight improvement in wave 3 (September/October 2021). Similarly, the prevalence of low HRQoL increased in the first two waves and improved slightly in wave 3, but was still high.

As far as positive changes experienced by their children during the Corona crisis, parents reported five themes that were found to correspond to Tedeschi and Calhoun’s (1995) PTG factors. Surprisingly, however, the spiritual and existential growth domain described by Tedeschi and Calhoun (1995) did not appear in our data. One possible explanation could be related to our highly secularised European world. In fact, it is not entirely clear to what extent the religious dimension of PTG is relevant for very secular countries (Pargament et al., 2006). Our findings, however, could also be due to the fact that the PTG factors are highly intercorrelated (Silverstein et al., 2018). In this study, for example, the theme “values and virtues,” which was assigned to the PTG factor “appreciation of life,” could possibly also represent an aspect of spirituality. In fact, Tedeschi and Calhoun (1995) initially divided growth into three general domains: changes in the perception of self, changes in the experience of relationships with others, and changes in the general philosophy of life, with the latter comprising priorities, appreciation and spirituality. Only later did factor analysis yield a five-factor approach to PTG (Tedeschi and Calhoun, 1996). Regarding the evolution of PTG over the course of the pandemic, parents at t1 (March 2020) reported more perceived PTG than parents at t4 (December 2021). This could be due to the specific characteristics of this crisis, e.g., the *creeping* aspect of a long-lasting crisis that is slow to subside and that is accompanied by frustration. Muñoz-Fernández and Rodríguez-Meirinhos (2021), for example, reported moderate to high levels of frustration in Spanish adolescents, which were associated with adolescents’ main concerns, online peer activities, maintenance of routines, and optimism. The association between PTG and better mental health is consistent with previous studies that reported positive associations with positive mental health factors, such as quality of life, and negative associations with psychopathology (e.g., Cann et al., 2010; Meyerson et al., 2011; Teodorescu et al., 2012).

In terms of sociodemographic and COVID-19-related variables affecting children’s mental health, male gender was associated with more aggressive behaviour. This finding confirms the normative difference in the levels of externalising problems reported by and about males and females as found in previous research, i.e., boys tend more toward externalising problems (which include aggressive behaviour) than girls (Crick and Zahn–Waxler, 2003). COVID-19-studies found similar results, i.e., boys show more externalising problems than girls (e.g., Giannotti et al., 2021; Schmidt et al., 2021).

Geographical location (urban/rural) and region (North/South Tyrol) had no significant impact on children’s mental health and HRQoL. Indeed, the COVID-19-studies examining the impact of geographical location (urban/rural) on children’s mental health do not show a consistent picture, with some identifying urban living as a risk factor (Duan et al., 2020), others identifying rural living as a risk factor (Hine et al., 2020; Zhou et al., 2020; Xu et al., 2021), and others finding no significant difference (Sarkadi et al., 2021). It should also be noted that North and South Tyrol are quite rural areas and the largest cities (Innsbruck in North Tyrol and Bolzano in South Tyrol) have around 130,000 and 100,000 inhabitants respectively. With regard to the region, i.e., North and South Tyrol – two bordering and similar regions, but belonging to two different states that have taken different Corona containment measures – our results are in line with those of Vasileva et al. (2021). Vasileva et al. (2021) conducted a mixed-methods study with 399 preschool children aged 3–5 years. Whilst discussing their results the authors stated that young children in Australia (their own study) and Spain (Idoiaga Mondragon et al., 2021) had similar concerns, although containment measures and objective levels of COVID-19 exposure were different in these countries.

This leads us to a particularly noteworthy result from this study: mental health and the HRQoL of Tyrolean children were not shown to be significantly impacted by actual exposure, while subjective threat experience proved to be the best predictor of children’s mental health and HRQoL (medium effect for posttraumatic stress symptoms, small effects for all other outcomes). Although some studies on children’s mental health during the COVID-19 pandemic identified exposure as a risk factor (Dönmez and Uçur, 2021; Luijten et al., 2021), this finding is not so clear-cut, and our study found pandemic exposure to have no impact on children’s mental health. This could be because only a small percentage of participating families were exposed to the most traumatic aspects of the pandemic (e.g., death of a close relative or hospitalisation due to COVID-19). That said, previous pandemics have shown that people do not necessarily need to live in areas with high rates of infection to experience stress (Blendon et al., 2004). Shanahan et al. (2020) found that most health risks to oneself or loved ones due to COVID-19 had no association with emotional stress (perceived stress, internalising symptoms, and anger). In line with our results, Shanahan et al.’s (2020) study found that the pre-pandemic distress, secondary consequences of the pandemic (e.g., lifestyle and economic disruption) and pre-pandemic social stressors were more strongly and consistently associated with distress of Swiss young adults aged 22 years during the COVID-19 crisis than exposure to virus-related health risks. Zhang et al. (2020) even found a negative association between the level of exposure to COVID-19 and mental health problems, confirming an effect called *Psychological Typhoon Eye*. This effect has been detected after different traumatic events (e.g., Wenchuan earthquake, SARS epidemic) and is explained by immunisation theory, cognitive dissonance theory and the gap between experiencing/involvement and imaging, wherein directly affected people at the centre of the event have a more accurate assessment of the risk based on real experiences and involvement. In Zhang et al.’s (2020) online study with 3,459 Chinese participants, perceived threat mediated the positive relationship between exposure level and mental health problems. Factors such as media coverage and perceptions of risk can be crucial for mental health (Blendon et al., 2004; Otto et al., 2007; Bhushan et al., 2022). Liu et al. (2020) found the concerns about the threat posed by COVID-19 to life and health was the only significant predictor of somatic symptoms in Chinese primary school children, which is consistent with our results that threat experience was the best predictor of children’s mental health outcomes. Research on other types of traumatic events and previous disasters has clearly shown that the risk of psychopathology is particularly high in relation to subjective experiences (Lonigan et al., 1991; Danese and Widom, 2020).

In addition to threat experience and perceived PTG, economic disruption was a significant predictor of children’s mental health and HRQoL. This result is consistent with other findings about children’s mental health (e.g., Reiss, 2013), even in times of COVID-19 (e.g., Fong and Iarocci, 2020; Ravens-Sieberer et al., 2021).

Finally, another important factor is the age of the children: the proportion of clinical classifications was higher among schoolchildren than preschool children in terms of internalising problems and aggressive behaviour, respectively. Additionally, our results show that threat experience was higher in older (schoolchildren) than in younger children (preschool children) and that threat experience among older children was higher at t4 than threat experience among older children at t1 (this was not true for younger children). Cost et al. (2021) investigated 1,013 parents of 6- to 18-year-old children, and 385 children/adolescents aged 10–18 years and found that deterioration in children’s mental health was age-dependent: deterioration in depression was highest among 10–12-year-old children while deterioration in anxiety and irritability was highest among 6–9-year-old children. Deteriorations in attention, hyperactivity, and obsessiveness/compulsivity were most common among adolescents. Preschool children were less likely to show deterioration in anxiety, irritability, and hyperactivity, displaying the lowest rates of deterioration and often the highest rates of improvement across all age groups. Based on these results, Cost et al. (2021) hypothesised that the impact of the pandemic on mental health would be greater for schoolchildren, for example in relation to the loss of daily routines (Brazendale et al., 2017). The loss of in-person classroom teaching may have had an additional negative impact on the mental health of schoolchildren (Hawrilenko et al., 2021). In terms of threat experience, older children participate more actively as individuals in society, and therefore have more of an opportunity to observe the effects of the pandemic. Developmentally, children typically do not understand key aspects of death (e.g., permanent/irreversible) until around 4–5 years of age (Panagiotaki et al., 2018), thus younger children will be less likely to perceive COVID-19 as *life-threatening* in the same way as older children (De Young et al., 2021). Younger children may be protected by a lack of awareness, e.g., because they cannot assess potential future consequences, whereas older children are all too aware of the harm (Masten and Motti-Stefanidi, 2020).

### Practical and Research Implications

The word *crisis* (Greek *krísis*) means *a crucial or decisive point or situation, a turning point*. A crisis is a turning point in the sense that it can aggravate problems, but at the same time a crisis can also be seen as a challenge and turned into an opportunity. In order to turn the current crisis into an opportunity with regard to children’s mental health, (a) the predictors discussed above can serve as a basis for assessing children at risk, and (b) the sustainability of PTG in children should be further promoted. It is important to monitor children for increased distress and where necessary, direct them to early intervention and treatment programs. Regarding the sustainability of PTG, there are several ways to facilitate PTG in children who have experienced stressful and traumatic situations, for example deliberate, constructive rumination; positive future expectations, hope and optimism; active coping; and social support (Kilmer et al., 2014).

Regarding exposure and threat, this study showed that subjective threat experience was the best predictor of poor mental health, and that threat experience was especially high among children aged 7–13. With respect to pandemic exposure, the picture was somewhat different: there was no significant association between pandemic exposure and mental health. Based on these results, parents, teachers, therapists, and other caregivers should pay special attention to children’s subjective threat experience (e.g., through child-appropriate conversations to help them understand the situation). In this context, the content and extent of media consumption are also crucial, as media coverage plays an important role in the development of worrisome thoughts (Vasileva et al., 2021). As far as politics and society is concerned, families’ financial situations must be monitored, as this study showed that financial disruption (one sixth of the families surveyed stated financial difficulties due to Corona) was a predictor of reduced mental health and HRQoL in children.

Finally, longitudinal research on the consequences of COVID-19 is needed to (a) be able to draw causal conclusions, (b) monitor children’s mental health, (c) observe the development of possible long-term pathologies and (d) be able to counteract them as early as possible. Further research is also needed to assess whether and for which children the threat experience and associated worries persist even in the absence of a current threat.

### Limitations

Limitations of the study include the fact that our sample was opportunistic and not representative. Those who were acutely affected by COVID-19 (or whose family members were) may not have participated. Therefore, particularly vulnerable groups may be underrepresented in our sample and our findings cannot be extrapolated to the general population. Furthermore, the findings are of cross-sectional nature, i.e., there were multiple measurement points during the pandemic, but different parents participated at each time point (1,972 parents participated once, 312 two or more times) and the cross-sectional design allows no causal conclusion. In addition, the retrospective assessment of mental health problems (t1) may have led to a distorted perception of symptoms, as the strictest lockdown (March 2020) was followed by a relaxation in June 2020, when the first measurement time point took place. Apart from this, children’s mental health, HRQoL, and PTG were assessed from parents’ perspective: children’s own assessments may differ from that of their parents (e.g., Jensen et al., 1999). Given that problem-scores tend to be higher in self-reports than in parent-reports (Rescorla et al., 2013), parents might have underreported their children’s mental health problems in this study. Also with regard to the PTG results, data from children’s own perspectives would be valuable to provide a completer and more differentiated picture. Finally, it would have been desirable to examine parents’ mental health as well. Scholarly literature shows that parents’ own symptomatology is strongly related to how they estimate their children’s symptoms (Exenberger et al., 2019).

### Conclusion

The COVID-19 pandemic led to children being burdened by negative effects on their mental health. Threat experience, financial disruption, and perceived PTG turned out to be significant predictors of children’s mental health and HRQoL. Targeted support for vulnerable children may prevent the longer-term development of psychopathologies and contribute to psychosocial resilience in society. Moreover, sustainable promotion of children’s PTG can also contribute to children’s mental health and could potentially even enable us to turn this crisis into an opportunity.

## Data Availability Statement

The raw data supporting the conclusions of this article will be made available by the authors, without undue reservation.

## Ethics Statement

The studies involving human participants were reviewed and approved by Medical University of Innsbruck. Written informed consent to participate in this study was provided by the participants.

## Author Contributions

AW, SE, KS, and BJ: conceptualisation. AW, SE, MS, KS, and BJ: methodology, and writing—review and editing. MS and AW: quantitative analysis. SE and AW: qualitative analysis. AW, SE, and MS: writing—original draft preparation. KS and SE: funding acquisition. All authors read and agreed to the published version of the manuscript.

## Conflict of Interest

The authors declare that the research was conducted in the absence of any commercial or financial relationships that could be construed as a potential conflict of interest.

## Publisher’s Note

All claims expressed in this article are solely those of the authors and do not necessarily represent those of their affiliated organizations, or those of the publisher, the editors and the reviewers. Any product that may be evaluated in this article, or claim that may be made by its manufacturer, is not guaranteed or endorsed by the publisher.
